# Nanoliposomal irinotecan plus fluorouracil and folinic acid as a second-line treatment option in patients with metastatic pancreatic ductal adenocarcinoma: a retrospective cohort study

**DOI:** 10.1186/s12885-021-08887-1

**Published:** 2021-11-03

**Authors:** Se Jun Park, Hyunho Kim, Kabsoo Shin, Tae Ho Hong, Ja Hee Suh, Myung Ah Lee

**Affiliations:** 1 Division of Medical Oncology, Department of Internal Medicine, Cancer Research Institute, College of Medicine, The Catholic University of Korea, Seoul St. Mary’s Hospital, 222 Banpo-daero, Secho-gu, Seoul, South Korea; 2 Division of Medical Oncology, Department of Internal Medicine, The Catholic University of Korea, St. Vincent’s Hospital, Suwon, South Korea; 3 Department of General Surgery, The Catholic University of Korea, Seoul St. Mary’s Hospital, Seoul, South Korea; 4 Department of Pathology, National Medical Center, Seoul, South Korea

**Keywords:** Pancreatic cancer, Liposomal irinotecan, Gemcitabine-refractory, Second-line treatment

## Abstract

**Background:**

According to the NAPOLI-1 trial, nanoliposomal irinotecan (nal-IRI) plus fluorouracil/folinic acid (5-FU/LV) showed improved overall survival compared to fluorouracil alone for patients with metastatic pancreatic cancer who were previously treated with gemcitabine-based therapy. In that trial, Asian patients had frequent dose modification due to haematological toxicity. There has been limited information on the clinical benefits and toxicity of this regimen in real-world settings. In this study, we assessed real-world experience of nal-IRI plus 5-FU/LV in patients with advanced pancreatic cancer after gemcitabine failure.

**Methods:**

We conducted a single institution, retrospective analysis of response, survival and safety in patients who had been treated with nal-IRI with 5-FU/LV. Patients with metastatic pancreatic ductal adenocarcinoma previously treated with gemcitabine-based therapy received nal-IRI (80 mg/m^2^) with 5-FU/LV every 2 weeks. Kaplan-Meier analysis was performed to obtain median progression free survival and median overall survival. The hazard ratio and 95% confidence interval (CI) were estimated using a stratified Cox regression model. A multivariate Cox proportional hazards regression model was used to identify the effects of clinical factors.

**Results:**

Fifty-one patients received nal-IRI plus 5-FU/LV between January 2015 and December 2020. The median age was 67 years, and males were 58.8%. A total of 40 (78.4%) and 11 (21.6%) patients had received one and two lines of prior chemotherapy before enrollment, respectively. Median progression-free survival was 2.8 months (95% CI 1.8–3.7) and median overall survival was 7.0 months (95% CI 6.0–7.9). Chemotherapy doses were reduced or delayed in 33 (64.7%) patients during the first 6 weeks and median relative dose intensity was 0.87. Thirty-six (70.6%) patients experienced grade 3 or 4 adverse events, most commonly neutropenia (58.8%). Most non-haematologic adverse events were under grade 2. Since the start of first-line chemotherapy, median overall survival was 16.3 months (95% CI 14.1–18.4).

**Conclusions:**

Nal-IRI plus 5-FU/LV seems to be effective, with manageable toxicities, following gemcitabine-based treatment in patients with metastatic pancreatic ductal adenocarcinoma. Nal-IRI plus 5-FU/LV following gemcitabine with nab-paclitaxel is a feasible sequential treatment option in patients with metastatic pancreatic cancer.

**Trial registration:**

Retrospectively registered.

## Background

Pancreatic ductal adenocarcinoma (PDAC) is a leading cause of cancer-related deaths worldwide, with more than 80% of patients presenting with either unresectable locally advanced or metastatic disease upon diagnosis [1, 2]. When curative resection is not possible, the prognosis is poor, with an overall 5-year survival rate of < 5% [3].

The development of new combination regimens including FOLFIRINOX (a combination of oxaliplatin, irinotecan, folinic acid and fluorouracil) and albumin-bound paclitaxel (nab-paclitaxel) with gemcitabine has improved the survival of patients with metastatic PDAC (mPDAC) [4, 5]. In clinical practice, for elderly patients or patients with relatively poor performance status (PS), nab-paclitaxel with gemcitabine is preferred to FOLFIRINOX due to toxicity concerns [6]. Although fluoropyrimidine-based combination regimens are recommended after gemcitabine-based treatment failure [7], more than half of patients who have previously been treated with gemcitabine-based therapy are not suitable for FOLFIRINOX due to poor general condition of their health [6, 8].

Nanoliposomal irinotecan (nal-IRI) consists of irinotecan free base encapsulated in liposome nanoparticles which maintain higher intra-tumoural levels of both irinotecan and SN-38 (the active metabolite of irinotecan) than conventional irinotecan [9]. In a phase III study (NAPOLI-1) of patients with mPDAC previously treated with gemcitabine-based therapy, nal-IRI combined with fluorouracil and folinic acid (5-FU/LV) showed superior survival to 5-FU/LV alone (6.1 months vs. 4.2 months; hazard ratio [HR], 0.67, *p* = 0.012); this combination also had a manageable toxicity profile [10]. Because of these results, nal-IRI combined with 5-FU/LV was approved by the U.S. FDA to be used as a subsequent therapy following gemcitabine-based treatment in patients with mPDAC.

Patients in the NAPOLI-1 trial had heterogeneity of clinical features such as previous anticancer therapy, and only half of the patients were treated with gemcitabine combination regimens. Real-world clinical data about efficacy, safety, and dose reduction of nal-IRI plus 5-FU/LV in patients with mPDAC who were previously treated with nab-paclitaxel with gemcitabine as first-line regimen are scarce [11, 12]. Therefore, clinical data are needed for nal-IRI plus 5-FU/LV in a changed clinical environment.

In this study, we retrospectively evaluated the efficacy and safety of nal-IRI plus 5-FU/LV in patients with mPDAC who failed treatment with nab-paclitaxel and gemcitabine. We also assessed the association between nal-IRI dose intensity and clinical outcomes. Furthermore, we investigated whether the use of nal-IRI with 5-FU/LV as a second-line treatment is a reasonable option as a continuum of care treatment algorithm in patients with mPDAC.

## Methods

### Patients

This was a single-institution, retrospective, observational analysis of patients diagnosed with mPDAC at the Catholic University of Korea, Seoul St. Mary’s Hospital from January 2015 to December 2020. We included patients aged 19 years or older with histologically confirmed recurrent or metastatic PDAC, who had failed gemcitabine-based treatment as first-line palliative therapy. Other key inclusion criteria were a Eastern Cooperative Oncology Group PS 0–2 and measurable or evaluable lesions according to the Response Evaluation Criteria in Solid Tumours (RECIST) version 1.1 criteria as well as adequate haematological (haemoglobin > 9.0 g/dL, white blood cell count > 4000/mm^3^, absolute neutrophil count > 1000/mm^3^, platelet count > 100,000/mm^3)^, renal function (creatinine < 1.5-fold the upper normal limit) and hepatic function (total bilirubin < 1.5-fold the upper normal limit, serum transaminase < 3-fold the upper normal limit).

### Treatment

Patients received intravenous infusion of nal-IRI at a dose of 80 mg/m^2^ (equivalent to 70 mg/m^2^ of irinotecan free base) over 90 min followed by folinic acid 400 mg/m^2^ over 30 min, then fluorouracil 2400 mg/m^2^ over 46 h, every 2 weeks. This treatment course was repeated until terminated because of disease progression, unacceptable toxicities, or patient’s refusal to continue.

Chemotherapy dose and schedule adjustments were allowed. Dose reduction was defined as a decrease of 15% in the chemotherapy dose relative to the standard regimen in the first three scheduled treatment cycles. Dose delays were defined as a delay of ≥7 days from the target date in the first three scheduled treatment cycles. Chemotherapy relative dose intensity (RDI) was defined as the ratio of the delivered dose intensity to the planned dose intensity expressed as percentage. Reduced RDI was defined as a RDI < 85% from standard dosing, because RDI < 85% has worse survival outcomes in various solid tumours [13].

We performed serial computed tomography scans and measured carbohydrate antigen 19–9 (CA 19–9) at baseline and every 6–8 weeks until disease progression. Radiographic tumour response assessment was analysed according to RECIST version 1.1. We assessed safety by grading adverse events according to the National Cancer Institute’s Common Terminology Criteria for Adverse Events, version 4.0.

### Statistical analysis

The objective response rate (ORR) represented the percentage of patients with a complete response (CR) or partial response (PR) and disease control rate (DCR) represented the percentage of patients with a CR or PR or stable disease (SD) among patients with measurable lesions. Progression-free survival (PFS) was defined as the time from the first dose of nal-IRI plus 5-FU/LV to the date of disease progression or death. Overall survival (OS) was estimated from the date of nal-IRI plus 5-FU/LV initiation to the date of death or last follow-up visit.

To assess the efficacy of the entire first-line and second-line treatment strategy as accurately as possible, we evaluated the PFS 2 and OS 2 in patients who received gemcitabine with nab-paclitaxel as first-line treatment followed by nal-IRI plus 5-FU/LV as second line therapy. PFS 2 was defined as the time from the initiation of gemcitabine with nab-paclitaxel to the date of disease progression on nal-IRI plus 5-FU/LV given after first disease progression, or death. OS 2 was defined as the time from the beginning of gemcitabine with nab-paclitaxel to the date of death from any cause.

Kaplan-Meier analysis was performed to obtain median OS and median PFS. The HR and 95% confidence intervals (CIs) for OS and PFS were estimated using a stratified Cox regression model. A multivariate Cox proportional hazards regression model was used to identify the effects of clinical factors on PFS and OS. All tests were two-sided and *p*-values < 0.05 were considered statistically significant. Statistical analyses were performed using IBM SPSS for Window version 24.0 (IBM SPSS Inc., Armonk, New York, USA) and GraphPad Prism version 8.0 (GraphPad Software Inc., San Diego, CA, USA).

## Results

### Patient characteristics

From January 1, 2015 to December 31, 2020, 51 patients were found to be eligible for this study. The baseline demographics and clinical characteristics are listed in Table 1. Median age was 67 years (range, 50–78) and 30 patients (58.8%) were male. The majority of patients presented with metastatic disease at the diagnosis and only nine patients (17.6%) had recurrent disease after curative surgery. Serum CA 19–9 levels were elevated in 42 (82.4%) patients at initiation of nal-IRI with 5-FU/LV treatment. Liver (80.4%), lung (31.4%), distant lymph node (31.4%) and peritoneum (29.4%) were the most frequent metastatic sites and 8 (15.7%) patients had more than three metastases.

**Table 1:** Baseline clinical characteristics

Variable	nal-IRI plus 5-FU/LV (***n*** = 51)
**Age**	
Median (Range)	67 (50–78)
**Gender**	
Male	30 (58.8%)
Female	21 (41.2%)
**Pancreatic tumor location**	
Head	19 (37.3%)
Body	18 (35.3%)
Tail	14 (27.4%)
**Disease status at start of nal-IRI**	
Recurrent	9 (17.6%)
Initially Metastatic	42 (82.4%)
**Baseline CA 19–9 level**	
Within normal range (< 40 U/mL)	9 (17.6%)
Above normal range (≥40 U/mL)	42 (82.4%)
**Site of metastatic lesions**	
Liver	41 (80.4%)
Lung	16 (31.4%)
Lymph node, Distant	16 (31.4%)
Peritoneum	15 (29.4%)
Bone	7 (13.7%)
**Measurable metastatic sites**	
1	19 (37.3%)
2	24 (47.0%)
≥ 3	8 (15.7%)
**Previous radiotherapy**	8 (15.7%)
**Previous surgery**	12 (23.5%)
**Previous lines of palliative chemotherapy**	
1	40 (78.4%)
2	11 (21.6%)
**Previous first-line palliative chemotherapy**	
Gemcitabine plus nab-paclitaxel	48 (94.1%)
Gemcitabine monotherapy	3 (5.9%)
**Previous irinotecan containing chemotherapy**	1 (2.0%)
**Previous 5-FU/LV containing chemotherapy**	13 (25.5%)

*Nal-IRI* nanoliposomal irinotecan, *5-FU/LV* fluorouracil/folinic acid, *CA 19–9* carbohydrate antigen 19–9

Forty patients (78.4%) had received one previous line of metastatic treatment, and 11 (21.6%) patients had previously received two lines of palliative chemotherapy. As first-line chemotherapy, gemcitabine plus nab-paclitaxel was given to most patients (94.1%), and only three patients (5.9%) had received gemcitabine monotherapy. Irinotecan and 5-FU/LV were previously administered in 1 (2.0%) and 13 (25.5%) patients, respectively.

### Treatment outcomes

A summary of treatment dose modifications is listed in Table 2. The median duration of exposure to nal-IRI plus 5-FU/LV was 1.9 months (range, 0.5–7.0) and the median number of cycles was four (range, 2–12). At the time of this analysis, seven patients (13.7%) were still undergoing nal-IRI plus 5-FU/LV treatment. Median RDI was 0.87 (range, 0.54–1.00) and 15 patients (29.4%) were treated with less than 85% RDI. Thirty patients (58.8%) had dose reduction and 13 patients (25.5%) had dose delay in first 6 weeks of treatment.

**Table 2:** Relative dose intensity, dose reduction and delay of the nal-IRI plus 5-FU/LV

	nal-IRI plus 5-FU/LV (***n*** = 51)
**Median duration of treatment, months (range)**	1.9 (0.5–7.0)
**Median cycles of treatment, n (range)**	4 (2–12)
**Median relative dose intensity, n (range)**	0.87 (0.54–1.00)
**RDI ≥ 85%, n (%)**	36 (70.6)
**RDI < 85%, n (%)**	15 (29.4)
**Dose reduction in first 3 cycles, n (%)**	30 (58.8)
**Dose delay in first 3 cycles, n (%)**	13 (25.5)

*Nal-IRI* nanoliposomal irinotecan, *5-FU/LV* fluorouracil/folinic acid, *RDI* relative dose intensity

**Fig. 1:**
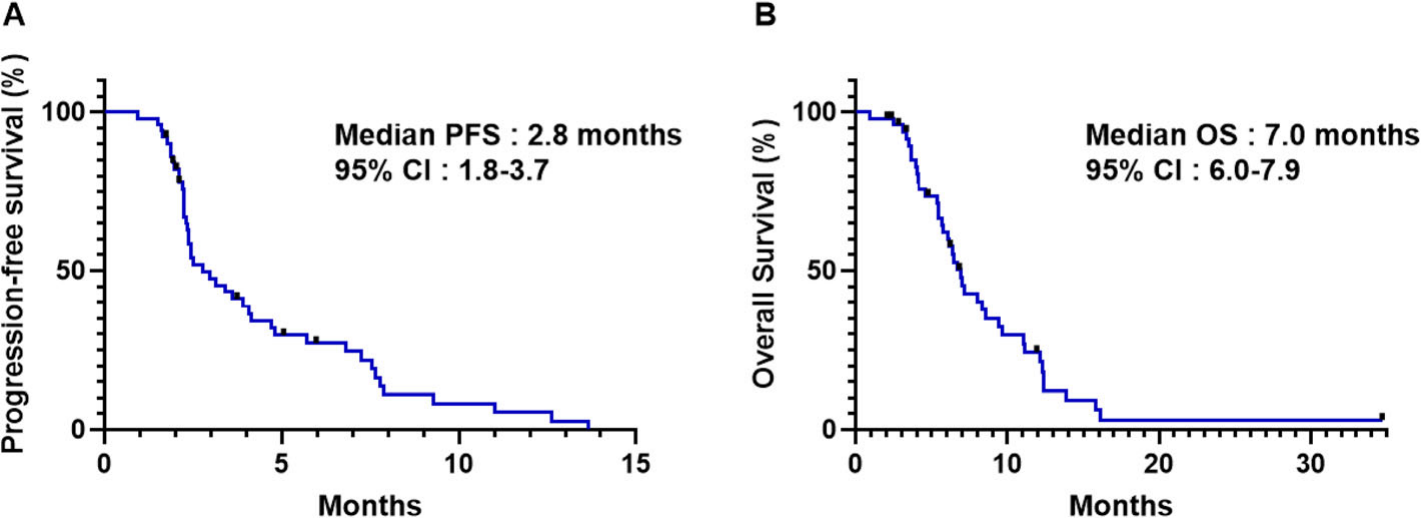
Kaplan-Meier analysis of (**A**) progression-free survival and (**B**) overall survival with nal-IRI plus 5-FU/LV

Effectiveness outcomes are summarised in Table 3. At the time of analysis, 44 patients (86.3%) had progressive disease. The response evaluation showed a partial response in three patients (5.9%), stable disease in 28 patients (54.9%), and progressive disease in 20 patients (39.2%). The ORR and DCR was 5.9 and 60.8%, respectively. The survival analysis was based on 39 (76.4%) deaths with a cutoff date of March 31, 2021. Median PFS was 2.8 months (95% CI 1.8–3.7) and median OS was 7.0 months (95% CI 6.0–7.9) (Table 3 and Fig. 1). The 6-month PFS and OS rate was 27.2% (95% CI 15.3–40.6) and 62.2% (95% CI 46.3–78.0), respectively.

**Table 3:** Efficacy of treatment with nal-IRI plus 5-FU/LV

	nal-IRI plus 5-FU/LV (***n*** = 51)
**Best response**	
**Complete response**	0
**Partial response**	3 (5.9%)
**Stable disease**	28 (54.9%)
**Progressive disease**	20 (39.2%)
**Objective response rate**	3 (5.9%)
**Disease control rate**	31 (60.8%)
**Median PFS, months [95% CI]**	2.8 [1.8–3.7]
**6-month PFS, % [95% CI]**	27.2 [15.3–40.6]
**Median OS, months [95% CI]**	7.0 [6.0–7.9]
**6-month OS, % [95% CI]**	62.2 [46.3–78.0]

*Nal-IRI* nanoliposomal irinotecan, *5-FU/LV* fluorouracil/folinic acid, *PFS* progression-free survival, *OS* overall survival

### Multivariate analysis of prognostic factors

**Fig. 2:**
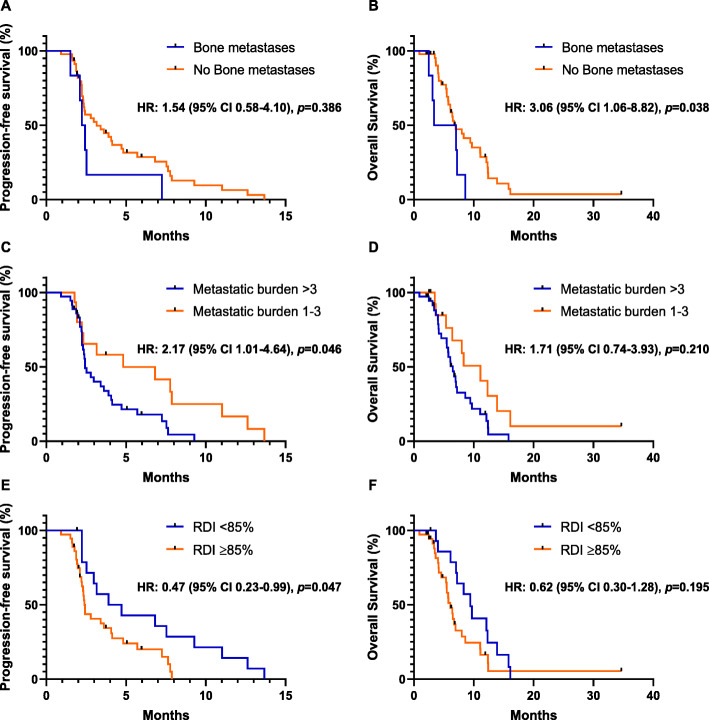
Subgroup survival analysis with nal-IRI plus 5-FU/LV. Progression-free survival and overall survival according to bone metastases (**A**, **B**), metastatic burden (**C**, **D**), and relative dose intensity (**E**, **F**)

Results of the multivariate analysis are shown in Table 4 with subgroups according to age (65 years older or younger), number of prior lines of chemotherapy, organ metastases (liver, lung, peritoneum and bone), metastatic burden (more or less than three metastases), RDI and pre-treatment neutrophil-to-lymphocyte ratio (NLR). Median PFS and OS did not differ significantly according to the number of prior lines of palliative chemotherapy (2 vs. 1) (*p* = 0.132 and *p* = 0.213, respectively). Also, PFS and OS were not affected by liver or lung metastases. Bone metastases were not related to PFS (HR = 1.54; 95% CI, 0.58–4.10; *p* = 0.386), but were significantly associated with worse OS outcomes (HR = 3.06; 95% CI, 1.06–8.82; *p* = 0.038) (Table 4. Figure 2A-B). Patients with high metastatic burden (metastases > 3) had worse PFS compared to patients with low metastatic burden (metastases 1–3) (HR = 2.17; 95% CI, 1.01–4.64; *p* = 0.046) without association with OS (HR = 1.71; 95% CI, 0.74–3.93; *p* = 0.210, Table 4. Figure 2C-D). Patients with reduced RDI (RDI < 85%) showed better PFS than patients with RDI ≥85% (HR = 0.47; 95% CI, 0.23–0.99; *p* = 0.047, Table 4. Figure 2E), however median OS did not differ significantly according to the RDI (HR = 0.62; 95% CI, 0.30–1.28, *p* = 0.195, Table 4. Figure 2F). No significant differences were evident in subgroups with high vs. low pre-treatment NLR (Table 4).

**Table 4:** Multivariate analysis of the clinical factors for PFS or OS in patients with mPDAC who received nal-IRI plus 5-FU/LV

Variables	PFS		OS	
	HR (95% CI)	*p* value	HR (95% CI)	*p* value
Age (≥65 vs. < 65 years)	0.80 (0.40–1.64)	0.554	1.09 (0.51–2.35)	0.824
Prior lines of chemotherapy (2 vs. 1)	1.82 (0.83–3.98)	0.132	1.78 (0.72–4.38)	0.213
Liver metastases	1.02 (0.40–2.58)	0.967	1.10 (0.49–2.52)	0.803
Peritoneum metastases	0.79 (0.36–1.72)	0.553	0.94 (0.42–2.08)	0.936
Bone metastases	1.54 (0.58–4.10)	0.386	**3.06 (1.06–8.82)**	**0.038**
Metastatic burden (> 3 vs. 1–3)	**2.17 (1.01–4.64)**	**0.046**	1.71 (0.74–3.93)	0.210
RDI (< 85% vs. ≥85%)	**0.47 (0.23–0.99)**	**0.047**	0.62 (0.30–1.28)	0.195
NLR (> 5 vs. ≤5)	0.82 (0.36–1.87)	0.635	1.47 (0.64–3.37)	0.364

*Nal-IRI* nanoliposomal irinotecan, *5-FU/LV* fluorouracil/folinic acid, *PFS* progression-free survival, *OS* overall survival, *HR* hazard ratio, *RDI* relative dose intensity, *NLR* neutrophil-to-lymphocyte ratio

### Safety

Table 5 summarises the treatment-related toxicity profiles. There were no treatment-related adverse events that resulted in death. Any-grade adverse events were observed in almost all patients (*n* = 50, 98%), and grade 3 or 4 adverse events were observed in 36 patients (70.6%). The most common treatment-related adverse events in patients receiving nal-IRI plus 5-FU/LV were anemia (*n* = 43, 84.3%), neutropenia (*n* = 43, 84.3%), nausea (*n* = 22, 43.1%), diarrhoea (*n* = 12, 23.5%) and fatigue (*n* = 11, 21.6%). Neutropenia was the most common grade 3 or 4 adverse event (*n* = 30, 58.8%). Febrile neutropenia was recorded in 4 patients (7.8%) and granulocyte colony stimulating factor was administered to 17 patients (33%).

**Table 5:** Toxicity profile during treatment

Adverse event	Any grade, n (%)	Grade 3–4, n (%)
All	50 (98.0)	36 (70.6)
Nausea	22 (43.1)	4 (7.8)
Vomiting	9 (17.6)	0
Diarrhea	12 (23.5)	3 (5.9)
Fatigue	11 (21.6)	2 (3.9)
Neutropenia	43 (84.3)	30 (58.8)
Febrile neutropenia	4 (7.8)	4 (7.8)
Anemia	43 (84.3)	14 (27.5)

### Survival outcome from beginning of the first-line treatment

**Fig. 3:**
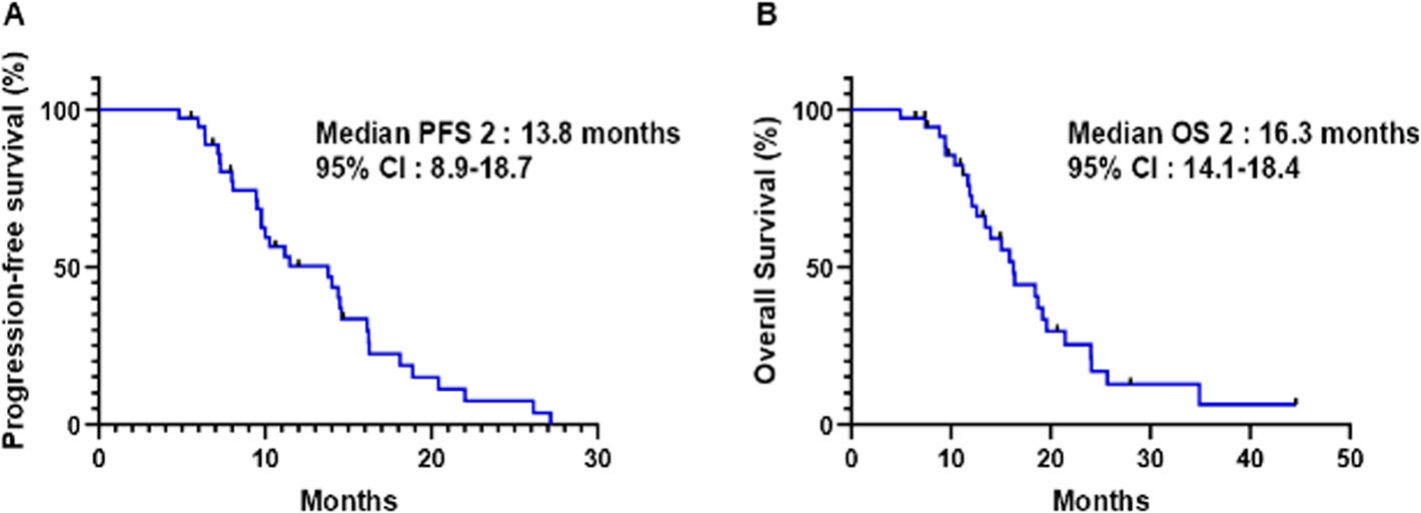
(**A**) Progression-free survival and (**B**) overall survival since the beginning of first-line chemotherapy

Of the total 51 patients, 37 patients (72.5%) were treated with gemcitabine with nab-paclitaxel as first-line chemotherapy followed by nal-IRI plus 5-FU/LV as second-line treatment. At a median follow-up of 13.3 months (95% CI 12.9–18.1), 31 patients (83.8%) had events of PFS 2 and 26 patients (70.3%) had events of OS 2. Median PFS 2 was 13.8 months (95% CI 8.9–18.7, Fig. 3A) and median OS 2 was 16.3 months (95% CI 14.1–18.4, Fig. 3B). The 1-year PFS 2 and OS 2 rates were 50.3% (95% CI 32.6–65.6) and 72.7% (95% CI, 54.0–84.8), respectively.

## Discussion

Since the results of the phase III MPACT trial and the phase III PRODIGE trial, gemcitabine with nab-paclitaxel or FOLFIRINOX has been used as first-line treatment for patients with mPDAC [4, 5]. As second-line treatment, for patients who have received prior gemcitabine-based therapy, fluoropyrimidine-based chemotherapy regimens are an acceptable option. On the other hand, gemcitabine-based treatment can be given to patients who have previously been treated with fluoropyrimidine-based therapy [7]. However, since there are debates about the optimal sequencing strategy for treatment of mPDAC, it is necessary to establish an optimal strategy through real-world clinical outcomes. This study evaluated effectiveness and safety of nal-IRI plus 5-FU/LV in patients with mPDAC following gemcitabine-based treatment in a real-world clinical setting. Furthermore, this is the first analysis to evaluate survival outcomes from the initiation of first-line treatment in patients with mPDAC who were treated with nal-IRI plus 5-FU/LV as second-line therapy after failure of first-line gemcitabine with nab-paclitaxel.

The characteristics of the patients in our study were comparable to those of patients enrolled in the NAPOLI-1 trial [10]. With respect to prior treatment, only 55% of patients in the NAPOLI-1 trial received gemcitabine combination treatment, but in our study, most patients (94.1%) were treated with gemcitabine with nab-paclitaxel as first-line treatment and only one patient was previously treated with a conventional irinotecan containing regimen. Patients who previously progressed on conventional irinotecan had poor survival outcomes with nal-IRI plus 5-FU/LV in prior studies [10, 11]. Compared to several other real-world studies of nal-IRI plus 5-FU/LV, the patients in our study were relatively homogenous in terms of their previous treatment history [11, 12].

Concerning the survival outcomes, the results of our study demonstrate real world evidence of treatment benefits with nal-IRI plus 5-FU/LV with similar outcome results that were reported in the NAPOLI-1 trial (median PFS 2.8 versus 3.1 months, median OS 7.0 versus 6.1 months). In our study, 6-month PFS and OS rates were 27.2 and 62.2%, respectively. These findings were consistent with the results of the NAPOLI-1 trial and prior real-world analyses of Asian populations [10, 14]. Based on these consistent clinical outcomes, despite differences in patient characteristics, nal-IRI plus 5-FU/LV showed real-world clinical benefits after gemcitabine with nab-paclitaxel failure.

With respect to dose modification, 33 (64.7%) patients experienced dose modification (dose reduction, *n* = 30; dose delay, *n* = 13) during the first 6 weeks. These findings are consistent with the results of the NAPOLI-1 study where 50 (60%) of 93 patients were treated with modified doses during the first 6 weeks [15]. Reduced RDI was expected to be associated with poor survival outcomes [13], but in our study, reduced RDI was not significantly associated with clinical outcomes; this is consistent with two previous studies [14, 15]. Patients with reduced RDI showed longer PFS, probably because patients who had been treated for a long period of time received more frequent modified doses of chemotherapy. Many patients with mPDAC deteriorate after first-line treatment failure, and frequent dose modification is necessary in order to reduce adverse events. Therefore, appropriate dose modification should be considered because dose modification was not significantly associated with survival outcomes. Moreover, according to the post hoc analysis of the NAPOLI-I study, Asian patients had more frequent haematological toxicities than Caucasian patients [16]; hence, dose adjustment should be considered in Asian patients.

We observed that several baseline characteristics were associated with survival outcomes through multivariate analysis. Patients with bone metastases had poor OS, which was consistent with the results of a previous real-world study [14]; in prior study, patients with liver metastases showed poor PFS [14]. Also, in analysis of NAPOLI-1 long-term survivals, patients who survived 1 year more were less likely to have had liver metastases [17]. However, in our study, no association between liver metastases and survival outcomes was observed. The subgroup analysis on metastatic burden indicates a better prognosis for the patients with fewer than three metastases. NLR at baseline was significantly associated with worse OS in the updated analysis of the NAPOLI-1 trial [17], but not in our study. Because the prognosis for patients with mPDAC remains poor, there is a critical need to evaluate a biomarker related to the efficacy of nal-IRI plus 5-FU/LV and select patients for optimal treatment based on prognostic biomarker.

Our results concerning adverse events are also comparable with the results previously reported in the NAPOLI-1 study, except that grade 3 or 4 diarrhoea was less frequently observed (5.9% vs. 13%), and grade 3 or 4 neutropenia (58.8% vs. 27%) was more frequently observed. The safety profile in our study was consistent with the results of the Asian subgroup analysis of the NAPOLI-1 trial including grade 3 or 4 diarrhoea (5.9% vs. 3.0%) and grade 3 or 4 neutropenia (58.8% vs. 54.5%) [16]. Most patients tolerated to treatment, with only 11% of patients discontinuing treatment because of any adverse events. According to an analysis of population pharmacokinetics of nal-IRI, the ethnic differences of adverse events could be associated with blood levels of unencapsulated SN-38 [18].

Although, gemcitabine with nab-paclitaxel or FOLFIRINOX are recommended as first-line treatment in patients with mPDAC, according to several real-world analyses, first-line FOLFIRINOX could only be used in 20–40% of mPDAC patients due to a higher incidence of haematological toxicity [19, 20]. Therefore, gemcitabine with nab-paclitaxel could be considered as first-line treatment for the other 50–60% patients with mPDAC. According to current National Comprehensive Cancer Network guidelines for the treatment of patients with mPDAC, 5-FU-based combination regimens are recommended as second-line therapy after gemcitabine-based treatment failure [7]. However, there is no universally accepted standard regimen for patients with mPDAC after gemcitabine with nab-paclitaxel. Nal-IRI plus 5-FU/LV could be considered in patients with poor PS, due to relatively manageable toxicities. Additionally, unlike the oxaliplatin plus 5-FU/LV regimen, nal-IRI plus 5-FU/LV is not associated peripheral neuropathy. Therefore, nal-IRI plus 5-FU/LV should be considered as a treatment option since patients who received gemcitabine with nab-paclitaxel, are more likely to have had peripheral neuropathy.

The two first-line treatment options, gemcitabine with nab-paclitaxel and FOLFIRINOX, have not been compared in the first-line setting. This means that currently the optimal first-line treatment and therapeutic sequence are unknown for patients with mPDAC. In our study population, the median OS from the start of first-line chemotherapy with gemcitabine plus nab-paclitaxel was 16.3 months. Excluding patients with ongoing of treatment using nal-IRI plus 5-FU/LV, 20 (45.5%) of 44 patients received best supportive care and 24 (54.5%) patients received a third line of chemotherapy after disease progression on nal-IRI plus 5-FU/LV. According to previous prospective studies, for patients who were treated with gemcitabine with nab-paclitaxel following FOLFIRINOX, the median OS from the initiation of first-line chemotherapy was 14.2–18.0 months [21, 22]. In a previous phase II study, patients treated with gemcitabine and nab-paclitaxel followed by modified FOLFIRNOX had a median survival time of 14.5 months [23]. Compared to these studies, sequential treatment of nal-IRI plus 5-FU/LV after gemcitabine with nab-paclitaxel appears to be a reasonable sequential treatment strategy. A comparative prospective randomised trial is needed to confirm the optimal sequential treatment strategy for patients with mPDAC.

There are some limitations in our study. First this was retrospective analysis conducted in a single centre. Second, the relatively small sample size limits the interpretation of subgroup analysis. Third, dose modification and treatment discontinuation were left to the discretion of the physicians, not according to any specified protocol. In addition, variability in response assessment intervals can have an effect on the results for PFS.

## Conclusions

Our experience demonstrated that nal-IRI plus 5-FU/LV was an effective and well-tolerated treatment following gemcitabine with nab-paclitaxel failure in a real-world clinical setting. Dose modification of nal-IRI did not adversely affect clinical outcomes. A strategy based on gemcitabine with nab-paclitaxel followed by nal-IRI plus 5-FU/LV is a feasible sequential treatment option in patients with mPDAC. Further studies are warranted to identify the optimal sequencing of systemic chemotherapy for patients with mPDAC.

## Data Availability

The datasets used in the current study are available from the corresponding author on request.

## Abbreviations

nal-IRI: Nanoliposomal irinotecan
5-FU/LV: fluorouracil/folinic acid
PDAC: Pancreatic ductal adenocarcinoma
mPDAC: Metastatic pancreatic ductal adenocarcinoma
PS: Performance status
RDI: Relative dose intensity
CA 19–9: Carbohydrate antigen 19–9
RECIST: Response Evaluation Criteria in Solid Tumours
ORR: Objective response rate
CR: Complete response
PR: Partial response
SD: Stable disease
DCR: Disease control rate
PFS: Progression-free survival
OS: Overall survival
NLR: Neutrophil-to-lymphocyte ratio
